# Vitiligo

**DOI:** 10.1111/ddg.15706_g

**Published:** 2025-08-11

**Authors:** Markus Böhm, Adrian Tanew

**Affiliations:** ^1^ Klinik für Hautkrankheiten Universitätsklinikum Münster, Deutschland; ^2^ Privatpraxis Wien Österreich

**Keywords:** gemeinsame Therapieentscheidung, Januskinase‐Hemmer, Komorbidität, Lichttherapie, Systemtherapie, topische Therapie, comorbidity, Janus kinase inhibitors, phototherapy, shared decision makting, systemic therapy, topical therapy

## Abstract

Vitiligo ist eine häufige Erkrankung des Pigmentsystems der Haut, die zur Zerstörung von Melanozyten führt. Während die segmentale Vitiligo (SV) pathogenetisch unzureichend verstanden ist, wird die nichtsegmentale Vitiligo (NSV) als Autoimmunerkrankung angesehen, bei der es auf dem Boden einer genetischen Disposition und erhöhten Vulnerabilität gegenüber Stressoren zur Aktivierung einer Melanozyten‐spezifischen CD8^+^‐vermittelten Immunantwort mit γ‐Interferon‐Signatur kommt. Vitiligo kann zu signifikanter Einschränkung der Lebensqualität führen und mit somatischen und/oder psychischen Störungen/Erkrankungen assoziiert sein. Frühzeitige Diagnose, korrekte Klassifikation, Erfassung von Krankheitsausdehnung und ‐aktivität, Krankheitslast und assoziierten Erkrankungen sind für ein holistisches Therapiemanagement essenziell. Die im gemeinsamen Entscheidungsprozess mit den Betroffenen definierten Therapieziele umfassen Stopp der Krankheitsprogression, Induktion von Repigmentierung, Vermeidung von Rezidiven und in seltenen Fällen Depigmentierung verbliebener Normalhaut. Die topischen Behandlungsoptionen beinhalten neben Kortikosteroiden und Calcineurininhibitoren mittlerweile den Januskinase‐Inhibitor Ruxolitinib als erste offiziell zugelassene Erstlinien‐Therapie für Patienten mit NSV ab 12 Jahren und Gesichtsbefall. Gezielte Lichttherapien, in Kombination mit topischen Kortikosteroiden oder Calcineurininhibitoren, kommen bei limitierter NSV oder SV in Frage. Bei ausgedehnter NSV bleibt die Ganzkörper Schmalband‐UVB Phototherapie therapeutischer Eckstein und kann bei rasch progredienter NSV mit einer oralen Kortison‐Minipulstherapie kombiniert werden. Unter den zukünftigen Therapieansätzen bei NSV sind systemische Januskinase‐Inhibitoren in der klinischen Forschung aktuell am weitesten fortgeschritten.

## EINLEITUNG

Vitiligo ist eine erworbene, üblicherweise chronische Erkrankung, die zu einem Verlust des Melaninpigments in der Haut führt. In den letzten Jahren sind bedeutende Fortschritte in der Aufklärung der Pathophysiologie dieser Erkrankung gemacht worden, die zu neuen vielversprechenden Therapieansätzen geführt haben.^1^ Neue epidemiologische Untersuchungen und Studien zur Krankheitslast und Häufigkeit assoziierter Erkrankungen erfordern zudem eine erweiterte Sicht auf die Krankheit Vitiligo mit dem Ziel einer holistischen und patientenorientierten Versorgung.^2^

### Epidemiologie

Je nach Studie und untersuchten Ländern (USA, Europa, Japan) liegt die Prävalenz zwischen 0,5% und 3,1%.^3^, ^4^ Eine kürzlich veröffentlichte Studie, die sowohl Daten einer großen gesetzlichen Versicherungsgesellschaft als auch primäre Daten einer großen Kohorte von durch Dermatologen untersuchten Probanden beinhaltete, beziffert die geschätzte Prävalenz in Deutschland zwischen 0,17% bz 0,77%.^5^

### Ätiopathogenese

**ABBILDUNG 1:**
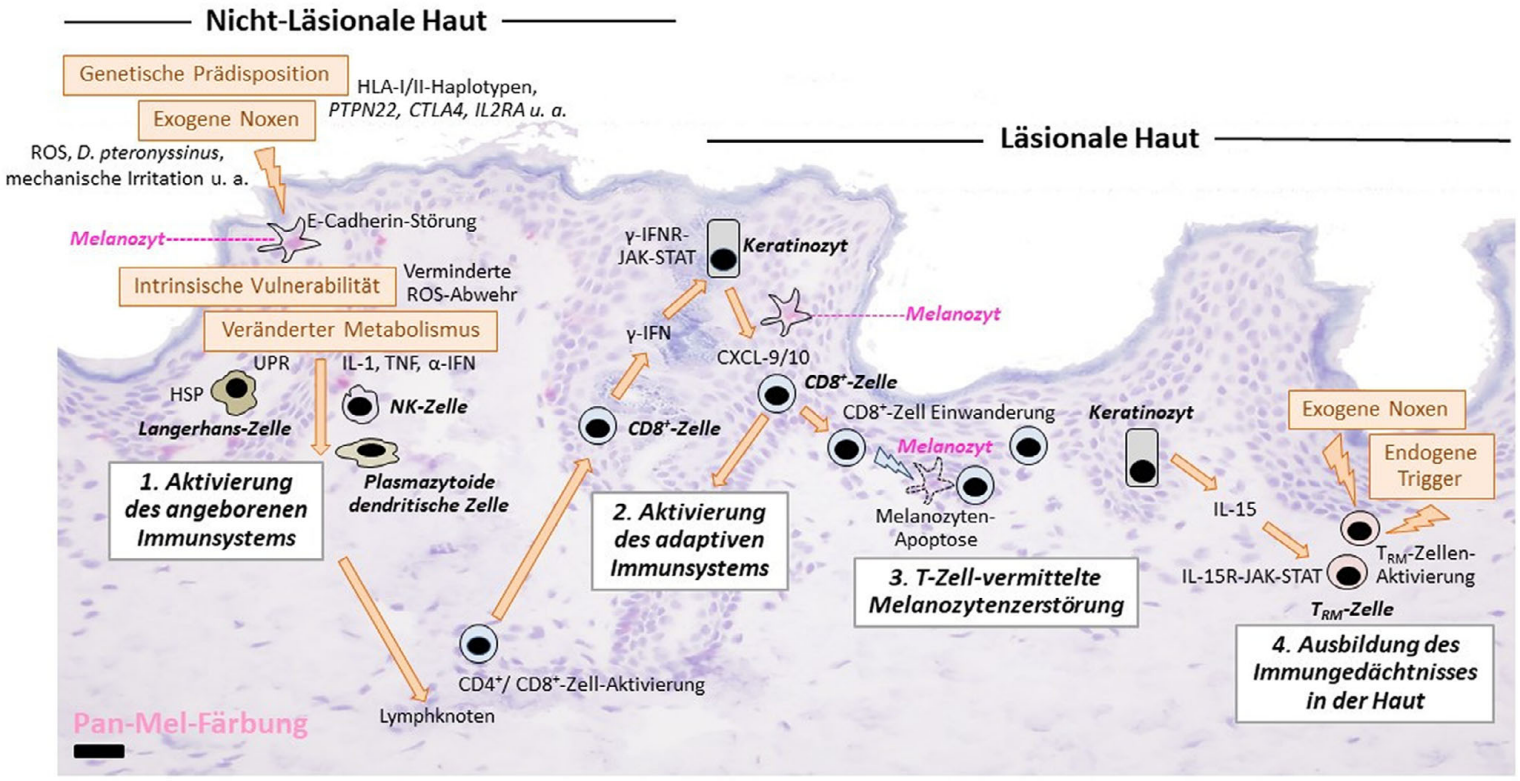
Pathophysiologie der nichtsegmentalen Vitiligo.

Der Hauptsubtyp der Vitiligo, die nichtsegmentale Vitiligo (NSV), wird heute übereinstimmend als Autoimmunerkrankung aufgefasst (Abbildung 1).^6^, ^7^ Grundlage für die Aktivierung der fehlgesteuerten zellulären Immunantwort gegen Melanozyten ist eine genetische Disposition, die molekulargenetisch durch eine Reihe genomweiter Assoziationsstudien bei Patienten mit NSV gut belegt ist.^8^ Dabei konnten mehr als 50 Empfänglichkeitsloci identifiziert werden, die das Risiko, eine NSV zu entwickeln, erhöhen. Zu den Empfänglichkeitsgenen zählen Gene, die den Stoffwechsel der Pigmentzellen orchestrieren, wie *TYR* oder *OCA2*, und dadurch für eine Melanozyten‐spezifische Antigenpräsentation relevant sind, sowie eine Vielzahl von Genen, die das angeborene und adaptive Immunsystem regulieren, wie *NLPR1, IRF3, PTPN1, CTLA4, IL2RA, HLA‐DRB1, FOXP3* oder *FASL*.^9^ Ein Teil dieser Gene wurde auch bei anderen Autoimmunerkrankungen als Empfänglichkeitsgene identifiziert und erklärt die Assoziation der NSV mit einer Reihe von autoimmunologischen Erkrankungen (siehe unten).

Der Hauptsubtyp der Vitiligo, die nichtsegmentale Vitiligo (NSV), wird heute übereinstimmend als Autoimmunerkrankung aufgefasst. Grundlage für die Aktivierung der fehlgesteuerten zellulären Immunantwort gegen Melanozyten ist eine genetische Disposition, die durch eine Reihe genomweiter Assoziationsstudien gut belegt ist.

Die polygenetische Disposition sowie intrinsische Anomalitäten der Melanozyten, unter anderem im Redoxsystem mit Störung der Abwehr oxidativen Stresses, bewirken eine vermehrte Vulnerabilität der Zellen gegenüber exogenen Noxen wie mechanische Irritation, pro‐oxidative Reize oder Hausstaubmilbenproteine.^10^, ^11^, ^12^ Melanozyten lösen sich hierbei aus dem epidermalen Zellverband (Melanozytorrhagie), was experimentell *ex vivo* reproduzierbar ist. Dieser Vorgang kommt durch Störungen der melanozytären Expression und Verteilung des Ankerproteins E‐Cadherin zustande und ist Matrixmetalloproteinase‐9 abhängig.^13^, ^14^, ^15^ Durch Expression von *danger‐associated molecular patterns* (DAMP) und Präsentation melanozytärer Antigene kommt es zur Anschaltung des angeborenen Immunsystems mit Aktivierung von plasmazytoiden dendritischen Zellen, NK‐Zellen und angeborenen lymphoiden Zellen.^6^, ^15^, ^16^, ^17^

In der zweiten Phase führt die Aktivierung des adaptiven Immunsystems zur Ausbildung einer „Immunsynapse“ zwischen aktivierten, einwandernden Melanozyten‐spezifischen CD8^+^ T‐Zellen mit γ‐Interferon (γ‐IFN)‐Signatur, epidermalen Keratinozyten und Melanozyten. Sekretion von γ‐IFN aus den CD8^+^ T‐Zellen bewirkt in epidermalen Keratinozyten eine Aktivierung des kanonischen JAK1/2‐STAT‐Signalwegs mit nachfolgender Sekretion von CXCL9/10.^18^ Diese Chemokine bewirken die Anlockung weiterer CD8^+^ T‐Zellen, so dass ein *circulus vitiosus* mit fortschreitender Zerstörung der Melanozyten und zunehmender Depigmentierung der Haut entsteht.^7^ Die durch CD8^+^ T‐Zellen vermittelte Immunantwort gegen Melanozyten lässt sich durch JAK1/2‐Hemmer im Tiermodell der Vitiligo unterdrücken und ist Rationale der aktuellen Therapiestrategien mit topischen und systemischen JAK‐Hemmern. Interessanterweise nehmen auch dermale Fibroblasten an der Immunpathogenese der Erkrankung teil und können durch regional unterschiedliche γ‐IFN‐Signaturen das symmetrische Befallsmuster der Vitiligoherde und die Therapieresistenz bestimmter Körperareale mit erklären.^19^

Sekretion von γ‐IFN aus den CD8^+^ T‐Zellen bewirkt in epidermalen Keratinozyten eine Aktivierung des kanonischen JAK1/2‐STAT‐Signalwegs mit nachfolgender Sekretion von CXCL9/10.

In der stabilen Phase der NSV werden residente CD8^+^ Gewebegedächtniszellen (*tissue resident memory cells*, TRM) für das kutane Krankheitsgedächtnis und die Rezidivneigung der NSV verantwortlich gemacht.^20^ Die Aktivierung dieser Zellen in zytotoxisch aktive Zellen ist Interleukin (IL)‐15 abhängig, wobei dieses Zytokin ebenso über den JAK‐STAT‐Signalweg intrazelluläre Signale übermittelt.^21^

In der stabilen Phase der NSV werden residente CD8^+^ Gewebegedächtniszellen (*tissue resident memory cells*, TRM) für das kutane Krankheitsgedächtnis und die Rezidivneigung der NSV verantwortlich gemacht.

Die Ätiopathogenese der segmentalen Vitiligo (SV) wird demgegenüber weniger gut verstanden. Vermutet wird ein lokalisierter immunpathogenetischer Mechanismus, der zu einer regional begrenzten und zeitlich limitierten Melanozytenzerstörung führt.^22^ Immunologisch existieren Unterschiede im Ausmaß von Stressproteinen und der Zahl zirkulierender regulatorischer T‐Zellen zwischen SV und NSV.^23^ Neben einem somatischen Mosaik sowie neurogenen Faktoren fanden sich kürzlich histopathologische, immunhistochemische und ultrastrukturelle Hinweise für die Gegenwart des Varizella‐Zoster‐Virus in aktiv sich ausbreitender SV.^24^

### Klinisches Bild

Klinisch kommt es bei der Vitiligo zu weißen Maculae, wobei die Körperhaare innerhalb der Vitiligoherde depigmentiert sein können (Leukotrichie, Poliosis). Leukotrichie in den Vitiligoherden wird als prognostisch ungünstiges Zeichen bewertet, da hier auch das follikuläre Stammzellreservoir der Melanozyten zerstört ist.^25^ Je nach Verhalten im Freien wird aufgrund der Reduktion oder des Fehlens des Melaninpigments in der Haut über eine vermehrte Sonnenlichtempfindlichkeit in den von Vitiligo befallenen Hautarealen geklagt. Bis zu 20% der Betroffenen geben läsionalen Pruritus an, der zumeist auf eine aktive Erkrankung hinweist.^26^ Bei aktiver Erkrankung finden sich isomorphe Reizeffekte, zum Beispiel nach mechanischer Irritation oder im Narbenbereich (Köbner‐Phänomen); die Ränder der Vitiligoherde können besonders bei dunkleren Hauttypen unvollständig depigmentiert sein (hypochrome Ränder) und mehrere Farbnuancen aufweisen (Trichrom‐Vitiligo). In selten Fällen kann es am Rand der weißen Flecken zu einem Erythem, teils sogar mit leicht tastbarer Infiltration, kommen (inflammatorische Vitiligo). Zeichen einer aktiven Vitiligo sind auch kleinste disseminierte Herde, sogenannte Konfetti‐Läsionen. Melanozytäre Naevi können periläsional depigmentieren (Halo‐Naevi), was aber auch unabhängig von einer Vitiligo auftreten kann.

In der aktuellen Klassifikation wird die Vitiligo in drei Hauptformen mit Untereinheiten eingeteilt. Der überwiegende Teil aller Patienten lässt sich der NSV zuordnen.

**ABBILDUNG 2:**
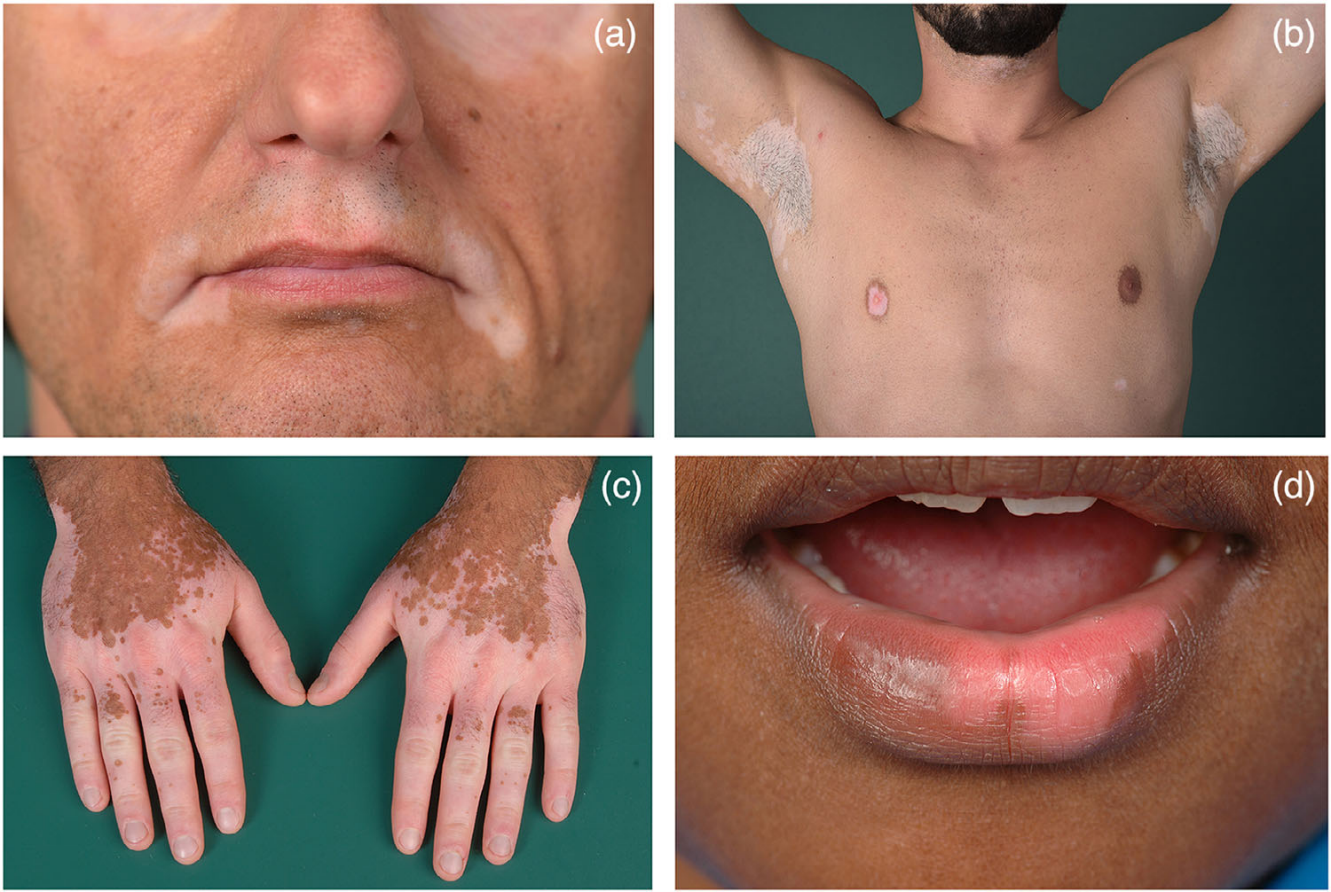
Klinisches Bild der nicht‐segmentalen Vitiligo. Symmetrischer Befall mit weissen Maculae periorifiziell im Gesicht (a), am Rumpf und in den Achseln (b), an den Extremitäten inklusive der Hände (c) und an der Schleimhaut (d).

In der aktuellen Klassifikation wird die Vitiligo in drei Hauptformen mit Untereinheiten eingeteilt (Tabelle 1).^27^ Der überwiegende Teil aller Patienten lässt sich der NSV zuordnen, deren gemeinsames Merkmal symmetrisch verteilte weiße Flecke mit Prädilektion im Gesicht, vor allem periorifiziell, und im Genitoanalbereich, an den Streckseiten der Extremitäten inklusive der Hand‐ und Fußgelenke, der Hand‐ und Fußrücken sowie der dorsalen Finger und Zehen ist (Abbildung 2a–).

**TABELLE 1:** Subtypen der Vitiligo.

**Nichtsegmentale Vitiligo (NSV)** Akrofazial Mukosal (mehr als eine Stelle) Generalisiert Universal Gemischt (NSV assoziiert mit SV) Seltene Varianten: ‐*Hypochrome Vitiligo* ‐*Follikuläre Vitiligo* ‐*Vitiligo punctata* **Segmentale Vitiligo (SV)** Unisegmental Bisegmental Polysegmental **Nicht klassifizierbare/determinierte Vitiligo** Fokal (eine Stelle) Mukosal (eine Stelle)

**ABBILDUNG 3:**
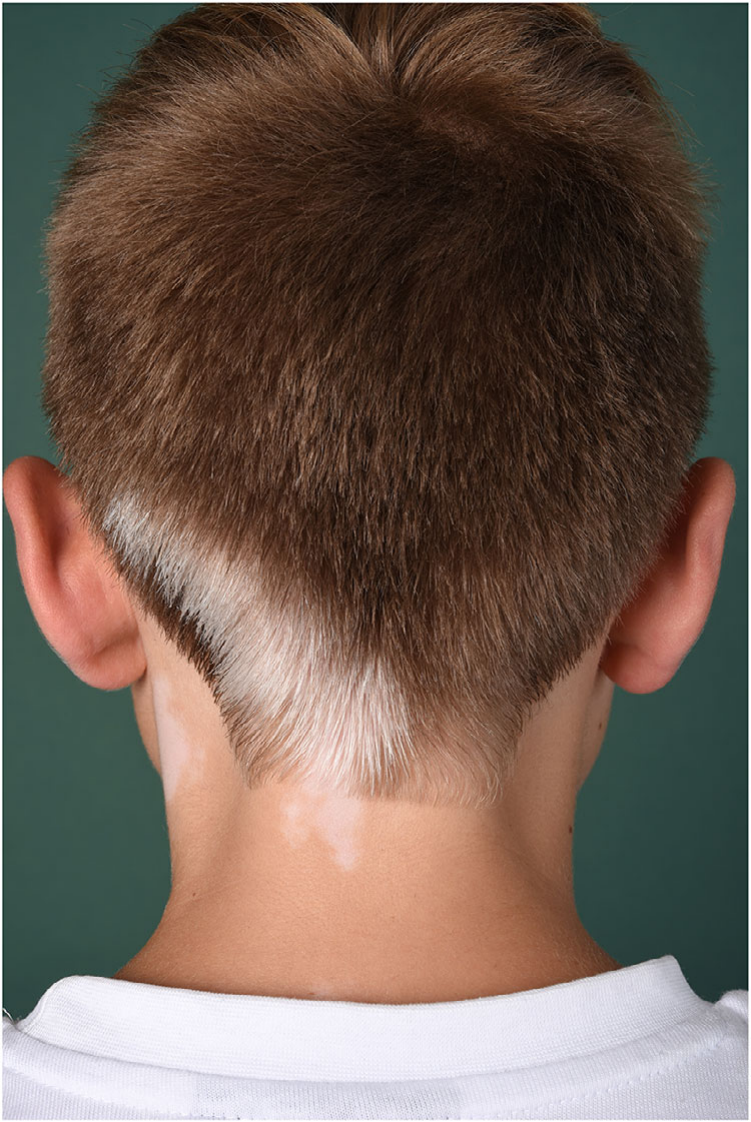
Klinische Präsentation der segmentalen Vitiligo. Unilateral verteilte weisse Flecke im Okzipitalbereich mit Befall der Dermatome C2‐C4 und mit Leukotrichie.

Die weit seltenere SV macht 5%–16% aller Vitiligofälle aus^28^, ^29^ und zeichnet sich durch einseitig lokalisierte weise Flecken mit frühzeitiger Leukotrichie aus. Uni‐, bi und polysegmentaler Befall kommt vor (Abbildung 3). Gleichzeitiges Auftreten unilateraler und symmetrisch verteilter weißer Flecken wird als gemischte Vitiligo bezeichnet, die aufgrund ihrer Prognose der NSV zugeordnet wird.

Davon abzugrenzen ist die nicht‐klassifizierbare (nicht determinierte) Form mit einzelnen Herden am Körper oder an der Schleimhaut, die sich im Verlauf von 1–2 Jahren durch Hinzukommen weiterer Herde als SV oder NSV demaskieren können (Tabelle 1). Zu den seltenen Sonderformen der NSV werden die follikuläre Vitiligo, die hypochrome Vitiligo bei Patienten mit dunkler Hautfarbe (nicht zu verwechseln mit den hypochromen Rändern als Zeichen einer aktiven Vitiligo) und die Vitiligo punctata gezählt (Tabelle 1).^27^

Die klinische Unterteilung in NSV und SV ist wichtig, da die NSV einen unvorhersehbaren Verlauf hat, mit anderen entzündlichen Krankheiten assoziiert ist (autoimmune Vitiligo) und häufig Rezidive zeigt.^30^ Während die NSV eine bimodale Altersverteilung mit Gipfeln im Alter von 10,3 und 34 Jahren hat,^31^ tritt die SV oftmals im Kindesalter auf. Leukotrichie tritt frühzeitig auf. Sie SV ist typischerweise nicht mit anderen Autoimmunerkrankungen assoziiert und kommt üblicherweise spontan nach 1–2 Jahren zum Stillstand.^22^, ^30^

### Differenzialdiagnose

Sie umfasst je nach Typ der Vitiligo und Ausdehnung angeborene und erworbene lokalisierte, generalisierte und universelle Depigmentierungserkrankungen (Reduktion beziehungsweise Fehlen der Melanozyten). Tabelle 2 listet die Differenzialdiagnosen auf. Eine histologische Sicherung ist in Zweifelsfällen sinnvoll, zum Beispiel zur Abgrenzung der progressiven makulären Hypomelanose (im Wood‐Licht oft follikuläre orangefarbene Fluoreszenz) und anderer seltener Leukodermien. Eine abgelaufene Pityriasis versicolor mit persistierenden hyopomelanotischen Flecken (Pityriasis versicolor alba) kann mit einer Vitiligo verwechselt werden. Manchmal kann auch ein Melasma irrtümlich als ausgedehnte faziale Vitiligo mit Restpigmentierung fehlinterpretiert werden.

**TABELLE 2:** Differenzialdiagnosen der Vitiligo.

**Kongenital / genetisch / syndromal** Naevus anaemicus Naevus depigmentosus Piebaldismus Tuberöse Sklerose Klein‐Waardenburg‐Syndrom Hypomelanosis Ito Albinismus Hermansky‐Pudlak‐Syndrom Menkes‐Syndrom Griscelli‐Syndrom Ziprkowski‐Margolis‐Syndrom **Postinflammatorisch/postinfektiös** Pityriasis alba Pityriasis versicolor Sarkoidose Psoriasis Leishmaniasis Syphilis Lepra Onchozerkose **Neoplasma‐assoziiert** Melanom Mycosis fungoides **Idiopathisch** Progressive makuläre Hypomelanose Hypomelanosis guttata idiopathica **Medikamentös bedingt** Interferon Imiquimod Checkpoint‐Inhibitoren Alemtuzumab Imatinib Cyclin‐abhängige Kinasen 4‐ und 6‐Hemmer **Chemisch induziert** Phenole und Phenolderivate **Andere Ursachen** Lichen sclerosus et atrophicus Eruptive Hypomelanose

### Assoziierte Störungen und Erkrankungen

Vitiligo kann mit einer Reihe von Erkrankungen assoziiert sein, wobei somatische und psychische Komorbidität zu unterscheiden sind.

### Somatische Komorbidität

Vor dem Hintergrund der genetischen Prädisposition der NSV zu Autoimmunerkrankungen ist besonders bei diesem Vitiligo‐Subtyp eine erhöhte Prävalenz weiterer autoimmunologischer Erkrankungen zu erwarten. In den dazu vorliegenden Studien wurde aber nicht immer klar zwischen NSV und SV unterschieden. Laut einer kürzlich publizierten Übersicht fand sich bei Vitiligo‐Patienten im Vergleich zur Allgemeinbevölkerung am häufigsten eine erhöhte Prävalenz von Schilddrüsenerkrankungen, Alopecia areata und Psoriasis vulgaris.^32^ Eine US‐amerikanische Studie an 1098 Patienten mit Vitiligo und 10‐jähriger Nachbeobachtung ergab, dass 20% aller Patienten mit Vitiligo mindestens eine weitere Autoimmunerkrankung haben.^33^ Die am häufigsten assoziierten Autoimmunerkrankungen waren Schilddrüsenerkrankungen (12,3%), gefolgt von Alopecia areata (3,8%). Diese Ergebnisse unterstützen frühere Befunde aus Studien mit ethnisch europäischen Patienten mit generalisierter Vitiligo aus den USA und England (Schilddrüsenerkrankungen bei 17% der Untersuchten), während eine Studie aus Taiwan mit 14 883 Patienten nur eine marginal erhöhte Frequenz von Schilddrüsenerkrankungen nachwies.^34^ Eine Metaanalyse von 37 Studien und 78 714 Patienten mit Vitiligo ergab Prävalenzen mit einer gepoolten *Odds Ratio* (OR) von 3,93 (95%‐Konfidenzintervall [KI]: 2,23–6,93) für Schilddrüsenerkrankungen, von 5,88 (95%‐KI: 2,68–12,89) für autoimmune Thyreopathien, von 3,38 (95%‐KI: 2,97–4,96) für Thyreoidea Peroxidase (TPO)‐Antikörper und von 3,51 (95%‐KI: 2,35–5,26) für Thyreoglobulin (TG)‐Antikörper.^35^ Die Prävalenz von Thyreopathien und TPO‐Antikörpern war bei Patienten mit NSV signifikant höher als bei Patienten mit SV und zeigte auch eine Abhängigkeit von der Ausdehnung der NSV.

Vor dem Hintergrund der genetischen Prädisposition der NSV zu Autoimmunerkrankungen ist besonders bei diesem Vitiligo‐Subtyp eine erhöhte Prävalenz weiterer autoimmunologischer Erkrankungen zu erwarten.

Laut einer kürzlich publizierten Übersicht fand sich bei Vitiligo‐Patienten im Vergleich zur Allgemeinbevölkerung am häufigsten eine erhöhte Prävalenz von Schilddrüsenerkrankungen, Alopecia areata und Psoriasis vulgaris.

Epidemiologische Studien erhärten ebenso den Zusammenhang zwischen Vitiligo und Diabetes mellitus beziehungsweise Komponenten des metabolischen Syndroms. Eine systematische Übersichtsarbeit mit Metaanalyse von neun Fallkontrollstudien (insgesamt 15 657 Vitiligo‐Patienten) ergab sowohl eine Assoziation von Vitiligo mit Diabetes mellitus Typ I (gepoolte OR: 2,90; 95%‐KI: 1,53–5,48; p  =  0,001) als auch Diabetes mellitus Typ II (gepoolte OR: 2,37; 95%‐KI: 1,71–3,28; p < 0,001).^36^ Diese Ergebnisse werden durch eine weitere Metaanalyse zur Komorbidität von Komponenten des metabolischen Syndroms bekräftigt. Bei den 30 analysierten Studien (insgesamt 28 325 Patienten mit Vitiligo) fanden sich signifikante Assoziationen mit Diabetes mellitus (gepoolte OR: 3,30; 95%‐KI: 2,10–5,17) und Adipositas (gepoolte OR: 2,08; 95%‐KI: 1,40–3,11). Die gepoolte Prävalenz von arterieller Hypertonie lag bei den Patienten bei 19.0% (95%‐KI: 2,0%–36,0%).^37^

In manchen Fällen kann NSV eine Komponente autoimmun polyglandulärer Syndrome (APS) sein. Systematische Analysen großer Kohorten von Vitiligo‐Patienten zur Prävalenz von APS fehlen jedoch. APS3 und 4 sind wahrscheinlich am häufigsten.^38^

Interessanterweise zeigten kürzlich zwei epidemiologische Studien aus Brasilien und Taiwan erhöhte Raten von sensorineuraler Hörminderung bei Patienten mit Vitiligo.^39^, ^40^ In der umfangreichen taiwanesischen Studie mit 12 048 Patienten und 52 192 Kontrollen ergab sich ein 2,2‐fach erhöhtes Risiko, diese Hörstörung zu bekommen. Diese Daten müssen in anderen Populationen bestätigt werden.

### Psychische Komorbidität

Wie bei anderen Dermatosen kann Vitiligo zu signifikanter Stigmatisierung betroffener Patienten mit erheblicher Einschränkung der Lebensqualität (Quality of Life, QoL) führen.^41^ Dies ist wiederum Grundlage für die Entwicklung psychischer Störungen und Erkrankungen. Viele Studien belegen, dass die QoL, gemessen mit dem *Dermatology Life Quality‐Index* (DLQI) – das global am häufigsten verwandte *Patient‐Reported Outcome Measure* (PROM)^42^, bei Patienten mit Vitiligo eingeschränkt ist. Befall sichtbarer Hautareale (Gesicht und Hände), aber auch der Genitalregion, jüngeres Erwachsenenalter und Ausmaß der Krankheitsausdehnung in Prozent der Körperoberfläche (*body surface area*, BSA) sind mit höherem DLQI (höhere Beeinträchtigung der Lebensqualität) verbunden. Hautfarbe, Beziehungsstatus und geographische Region spielen ebenso eine Rolle.^42^, ^43^

Wie bei anderen Dermatosen kann Vitiligo zu signifikanter Stigmatisierung betroffener Patienten mit erheblicher Einschränkung der Lebensqualität führen.

Dass Vitiligo mit psychosozialer und psychischer Komorbidität verbunden ist, zeigen auch aktuelle systematische Übersichten und Metaanalysen.^44^, ^45^, ^46^, ^47^ Von 168 Studien, die in der jüngsten Übersichtsarbeit eingeschlossen waren, berichteten die meisten über depressive Symptome und depressive Störungen (41 Studien) sowie Angststörungen (20 Studien).^47^ Weitere festgestellte psychische Störungen waren Anpassungsstörungen (12 Studien), Suizidalität (8 Studien), Schlafstörungen (7 Studien), Zwangsstörungen (5 Studien) und somatoforme Störungen (3 Studien). Zudem wurden häufig psychosoziale Belastungen und Begleitsymptome beobachtet. Zehn Studien berichteten über Beziehungsprobleme inklusive sexueller Dysfunktion, acht Studien über Stigmatisierung. Des Weiteren wurde bei Vitiligo Patienten Vermeidungsverhalten (9 Studien), vermindertes Selbstbewusstsein (8 Studien), Wut (6 Studien), Alexithymie (4 Studien) und geringes Selbstwertgefühl (4 Studien) gefunden. Je nach Studiendesign und untersuchter geographischer Region fanden sich aber große Spannbreiten in den Häufigkeiten dieser psychosozialen und psychischen Komorbiditäten.^47^

### Diagnostik

**ABBILDUNG 4:**
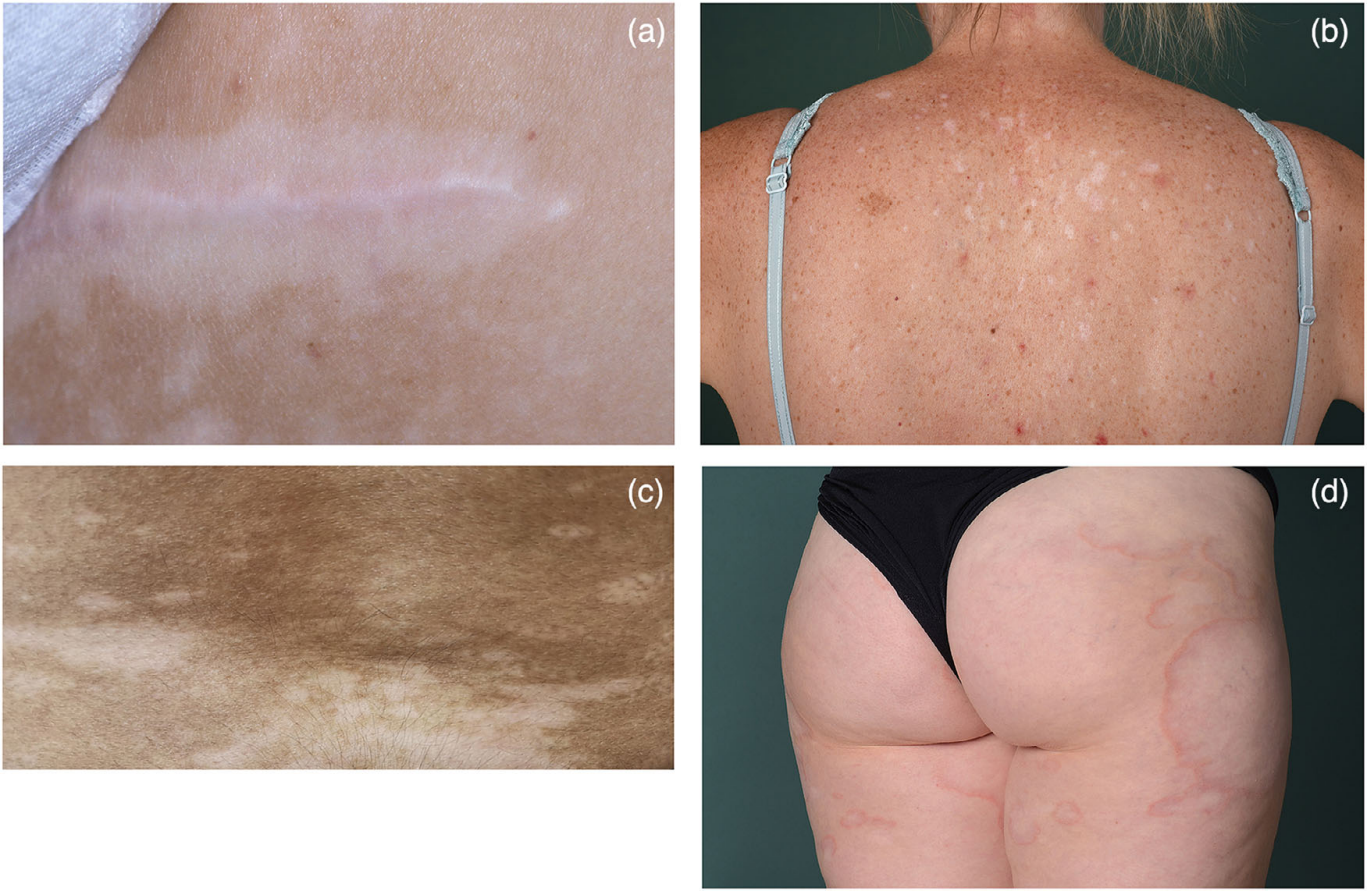
Klinische Aktivitätszeichen der Vitiligo. (a) Köbner‐Phänomen in einer Narbe, (b) Konfetti‐Läsionen; cave Verwechselung mit Lichen sclerosus et atrophicus, aber hier keine Konsistenzvermehrung, (c) hypochrome Ränder und (d) inflammierte Vitiligo (d).

Die Diagnose Vitiligo wird üblicherweise klinisch gestellt. Untersuchung mittels Wood‐Licht erleichtert das Erkennen von Vitiligoherden, besonders bei Betroffenen mit hellem Hauttyp oder Vorliegen von Konfetti‐Läsionen.^48^ Alle Patienten sollten auf klinische Zeichen einer aktiven Erkrankung (Köbner‐Phänomen, hypochrome Ränder, inflammierte Vitiligo und Konfetti‐Läsionen) (Abbildung 4) untersucht werden, wobei das Köbner‐Phänomen als validestes Kriterium einer aktiven Erkrankung identifiziert wurde.^49^ Fotographische Dokumentation kann die Einschätzung von Aktivität und Ausdehnung verbessern, wenn sie standardisiert im Verlauf durchgeführt wird.^50^

Die Diagnose Vitiligo wird üblicherweise klinisch gestellt. Untersuchung mittels Wood‐Licht erleichtert das Erkennen von Vitiligoherden, besonders bei Betroffenen mit hellem Hauttyp oder Vorliegen von Konfetti‐Läsionen.

Für die Bestimmung der Ausdehnung der Vitiligo, die im zeitlichen Verlauf einen Rückschluss auf die Krankheitsaktivität erlaubt, stehen validierte Scores zur Verfügung.

Für die Bestimmung der Ausdehnung der Vitiligo, die im zeitlichen Verlauf einen Rückschluss auf die Krankheitsaktivität (aktiv, nicht‐aktiv, stabil) erlaubt, stehen validierte Scores zur Verfügung (Tabelle 3).^51^, ^52^, ^53^, ^54^, ^55^ Die Einschätzung und Dokumentation der betroffenen Körperoberfläche in Prozent ist bei allen Messinstrumenten zentrales Element. Dies ist zudem wesentlich für die Therapieplanung. Die ganze Patientenhandfläche inklusive der volaren Fingerseiten entspricht 1% BSA, der Zeigefinger 0,1% BSA, das distale Segment des Zeigefingers 0,03% BSA.^56^ Eine allgemein anerkannte Einteilung der betroffenen Körperoberfläche in Schweregrade gibt es nicht; eine aus Patientensicht gewonnene Einteilung ist wie folgt: mild ≤ 1,05% BSA, moderat > 1,05–6,45% BSA und schwer > 6,45% BSA.^57^ Der *Vitiligo Area Scoring Index* (VASI) ist der meist verbreitete Score in klinischen Studien, er beinhaltet nicht nur die Einschätzung der befallenen Körperoberfläche, sondern auch das Ausmaß der Depigmentierung innerhalb der befallenen Hautareale.^51^ In den klinischen Studien wird der VASI oft separat nur für das Gesicht (F‐VASI) und den ganzen Körper (T‐VASI) angegeben. Der von der *Vitiligo European Task Force* (VETF) beschriebene Score ermittelt ebenfalls den Körperoberflächenbefall und Depigmentierungsgrad, erfasst aber auch zusätzlich die Krankheitsaktivität.^52^ Der *Vitiligo Extent Score* (VES) basiert auf Einschätzung des Körperoberflächenbefalles anhand von Vergleichsbildern^53^ und kann über eine App (https://www.vitiligo‐calculator.com/) sowohl vom Untersucher als auch von Patienten (*Self‐Assessment‐Vitiligo Extent Score*, SA‐VES) benutzt werden.^54^ VES und SA‐VES zeigten eine exzellente Korrelation miteinander.^54^

Aufgrund der Assoziation insbesondere der NSV mit autoimmunologischen Erkrankungen sollte bei Patienten mit Vitiligo und negativer Anamnese das Vorliegen einer latenten Autoimmunthyreoiditis ausgeschlossen werden.

**TABELLE 3:** Scores zur Messung der Vitiligoausdehnung.

Score	Akronym	Referenz
Vitiligo Area Scoring Index	VASI	Hamzavi, et al. 2004
Vitiligo European Task Force assessment	VETFa	Taieb, et al. 2007
Vitiligo Extent Score	VES	van Geel, et al. 2016
Self‐Assessment‐Vitiligo Extent Score	SA‐VES	van Geel, et al. 2017
Vitiligo Extent Score plus	VESplus	van Geel, et al. 2018

Aufgrund der Assoziation insbesondere der NSV mit autoimmunologischen Erkrankungen sollte bei Patienten mit Vitiligo und negativer Anamnese das Vorliegen einer latenten Autoimmunthyreoiditis durch die Bestimmung von Thyreoidea‐stimulierendem Hormon (TSH), TPO‐ und TG‐Antikörpern sowie Thyreotropin‐Rezeptor Antikörpern ausgeschlossen werden.^58^, ^59^, ^60^ Bei entsprechenden anamnestischen oder klinischen Hinweisen ist eine weiterführende Diagnostik zum Ausschluss weiterer Erkrankungen, beispielsweise des atopischen Formenkreises, Diabetes mellitus oder einer perniziösen Anämie, sinnvoll. Ein routinemäßiges breites Screening auf Autoantikörper wird nicht empfohlen.

Zum Screening der Lebensqualität und Krankheitslast betroffener Patienten mit Vitiligo wird in klinischen Studien zumeist der DLQI als PROM verwendet, obwohl er nicht spezifisch für Vitiligo ist. Vitiligo (Viti)QoL^61^ und die *Vitiligo‐Impact‐Patient‐Scale* (VIPs)^62^ sind demgegenüber als validierte PROM geeigneter. Die Anwendung dieser PROM vermittelt wichtige Informationen für die weitere Therapieplanung und etwaige Wahl supportiver Therapien (siehe unten).^58^, ^59^

### Therapiemöglichkeiten

**ABBILDUNG 5:**
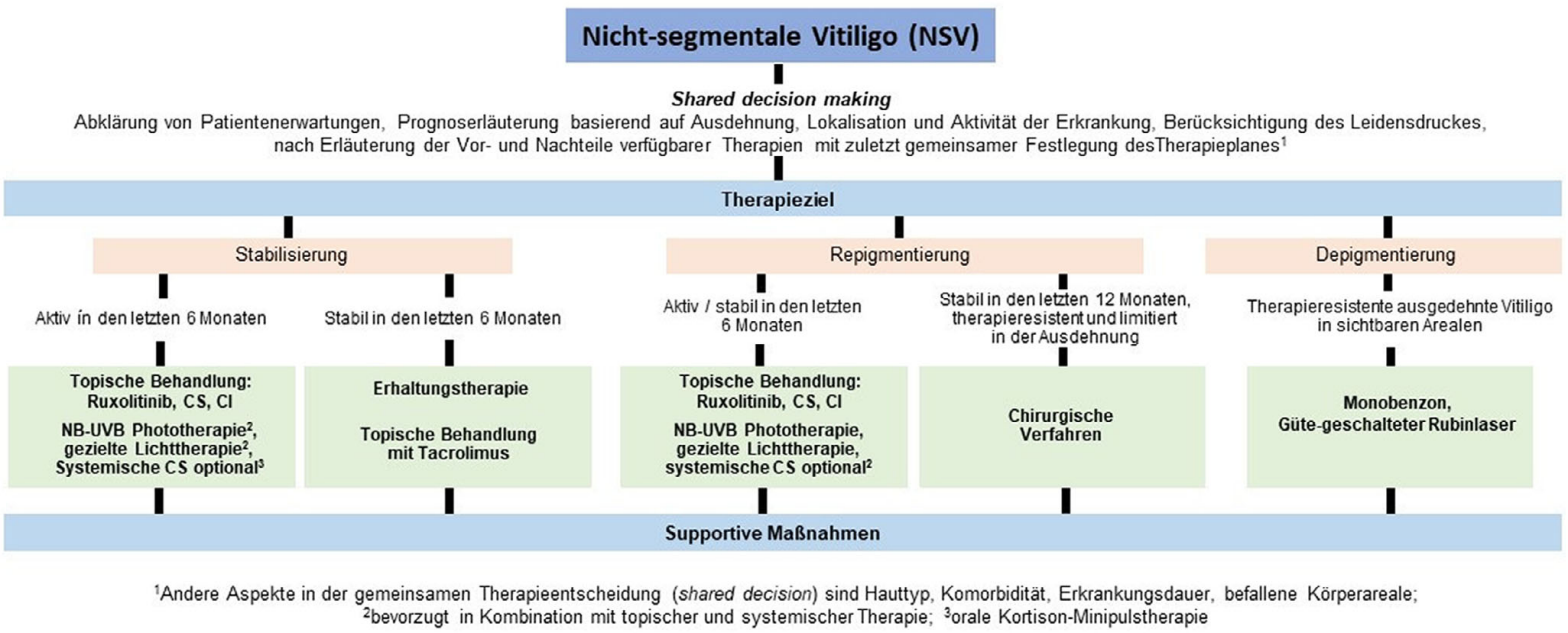
Vereinfachtes Therapieschema der häufigsten Form der Vitiligo, der nichtsegmentalen Vitiligo (NSV) (in Anlehnung an ^63^). *Abk*.: CS, Kortikosteroide; CI, Calcineurininhibitoren.

Die Therapie richtet sich nach dem Subtyp der Vitiligo, dem Alter und Hautphototyp nach Fitzpatrick, der Ausdehnung und Aktivität der Erkrankung, nach vorhandenen Komorbiditäten und dem individuellen Leidensdruck der Betroffenen.^58^, ^59^, ^63^ Gemeinsam mit den Patienten sollten die Therapieziele definiert werden: *(1)* Stopp der Progression der Erkrankung, *(2)* Induktion einer Repigmentierung *(3)* Erhalt der erzielten Repigmentierung (Stabilisierung). Nur in Ausnahmefällen kommt eine *(4)* Depigmentierung in Betracht (Abbildung 5). Da hier die Melanozyten der nichtläsionalen gesunden Haut dauerhaft zerstört werden sollen, ist dieses Ziel unseres Erachtens insbesondere bei europäischen Hauttypen nur in Einzelfällen als *ultima ratio* nach Versagen aller verfügbaren Therapien zu verstehen.

Gemeinsam mit den Patienten sollten die Therapieziele definiert werden: *(1)* Stopp der Progression der Erkrankung, *(2)* Induktion einer Repigmentierung *(3)* Erhalt der erzielten Repigmentierung (Stabilisierung). Nur in Ausnahmefällen kommt eine *(4)* Depigmentierung in Betracht.

## TOPISCHE THERAPIEN

### Topische Kortikosteroide

Aufgrund ihrer breiten, aber unspezifischen antiinflammatorischen Wirkung werden topische Kortikosteroide (*topical corticosteroids*; TCS) seit Jahrzehnten zur Behandlung der Vitiligo eingesetzt, um die Erkrankung zu stabilisieren und/oder Repigmentierung zu erzielen. Topische Kortikosteroide gelten hierbei als mögliche Erstlinien‐Therapie bei limitierter Ausdehnung und besonders bei extrafazialem Befall.^58^, ^63^ Das Rationale für eine Monotherapie mit TCS bei Vitiligo basiert auf älteren und meist kleineren Studien (< 100 Patienten), deren Design allerdings nicht dem heutigen Standard klinischer Studien entspricht und deren Ergebnisse mit denen der neuesten randomisierten kontrollierten Studien (RCT) nur bedingt vergleichbar sind. In einer Metaanalyse von Patienten mit lokalisierter Vitiligo (< 20% BSA) wurde ein gepooltes Ausmaß der erreichten 75%igen Repigmentierung bei TCS der Klasse IV (Clobetasol‐2‐propionat) von 55% errechnet, wobei kein signifikanter Unterschied zu Klasse‐III‐Präparaten wie Betamethason‐17‐Valerat bestanden bestand (56%).^64^ Mit in diese Metaanalyse eingeflossen sind jedoch auch Ergebnisse aus nichtrandomisierten kontrollierten Studien, aus Studien mit unterschiedlich starken TCS und solchen mit unterschiedlich langer Applikationsdauer (2–21 Monate). Realistischer erscheinen die Ansprechraten, die in neueren RCT gewonnen wurden, in denen TCS und topische Calcineurininhibitoren verglichen wurden (siehe unten). Repigmentierung durch TCS ist wie bei allen Vitiligo‐Therapien in erster Linie im Gesichts‐ und Halsbereich zu erwarten, während Stamm, Extremitäten und akrale Körperareale und solche mit Leukotrichie üblicherweise schlechter ansprechen. Die bekannten unerwünschten Wirkungen mittelstarker und starker TCS bei längerer Applikation (Atrophie, Teleangiektasien, Hypertrichose, akneartige Veränderungen) limitieren die Anwendung älterer TCS auf wenige Monate. Vorsicht ist besonders im Gesicht (vor allem im Lidbereich) und bei Kindern in den intertriginösen Bereichen (Absorptionsgefahr) geboten. Um unerwünschte Wirkungen zu vermeiden, sollten moderne Klasse‐III‐TCS mit geringem atrophogenen Risiko wie zum Beispiel Mometasonfuroat verwendet werden. Eine evidenzbasierte Datenlage zum optimalen Applikationsregime von TCS bei Vitiligo existiert aber nicht. Ein vorgeschlagenes Therapieregime besteht in einer Applikation für 3 Monate einmal täglich oder alternativ für maximal 6 Monate einmal täglich im Wechsel von 2 Wochen Behandlung und 2 Wochen Pause.^58^, ^63^ Bei Repigmentierung im Körperbereich ohne Nebenwirkungen kann diese Therapie aber auch verlängert werden.

Topische Kortikosteroide gelten als mögliche Erstlinien‐Therapie bei limitierter Ausdehnung und besonders bei extrafazialem Befall.

### Topische Calcineurininhibitoren

Die topischen Calcineurininhibitoren (TCI) Tacrolimus und Pimecrolimus gelten ebenfalls als Erstlinien‐Behandlung für erwachsene Patienten und Kinder mit limitierter Vitiligo, besonders im Gesicht‐ und Halsbereich sowie in den intertriginösen Bereichen, wo topische Kortikosteroide problematisch sind. Die repigmentierende Wirkung der TCI wird auf deren unspezifische Entzündungshemmung und auf einen migrationsfördernden Effekt auf Melanozyten zurückgeführt. Beide Substanzen sind jedoch nur für die atopische Dermatitis zugelassen, bei Vitiligo ist ihr Einsatz *off‐label* und damit prinzipiell nicht erstattungsfähig. Die repigmentierende Wirkung der topischen Kortikosteroide und TCI ist im Gesichtsbereich ähnlich (hier sind TCS aber wegen der unerwünschten Wirkungen nicht zu empfehlen), an anderen Körperstellen scheinen Kortikosteroide den TCI in der Wirksamkeit überlegen.^65^, ^66^, ^67^ In einer Metaanalyse wurde bei 55% der Patienten eine Repigmentierung von mindestens 25% und bei 18,1% eine Repigmentierung von mindestens 75% beobachtet. Allerdings hatten die Patienten im Median lediglich über 3 Monate eine Monotherapie mit TCI erhalten.^68^ Bei Kindern zeigte sich im Gesicht‐ und Halsbereich bei 35,4% eine Repigmentierung von mindestens 75%. Okklusion kann den Effekt einer Monotherapie mit TCI verstärken.^69^ Zweimalige Applikation für mindestens 6–9 Monate wird empfohlen,^70^ bei Ansprechen und fehlenden Nebenwirkungen können TCI aber auch länger verabreicht werden. TCI eignen sich auch zum Erhalt der Pigmentierung nach erfolgreicher Behandlung. In einer kleinen RCT konnte die Applikation von 0,1%iger Tacrolimus‐Salbe zweimal wöchentlich über einen Zeitraum von 24 Wochen das Rezidivrisiko von 48,4% (Placebo) auf 26,8% reduzieren.^71^ Zu den häufigsten unerwünschten Wirkungen der TCI gehören Pruritus, brennende Sensationen und Rötungen im Applikationsbereich (unter anderem auch bei Alkoholgenuss).

Die topischen Calcineurininhibitoren Tacrolimus und Pimecrolimus gelten ebenfalls als Erstlinien‐Behandlung für erwachsene Patienten und Kinder mit limitierter Vitiligo, besonders im Gesicht‐ und Halsbereich sowie in den intertriginösen Bereichen.

### Topisches Ruxolitinib

Mit der europaweiten Zulassung des JAK‐1/2‐Inhibitors Ruxolitinib steht seit April 2023 erstmals eine offiziell zugelassene Erstlinien‐Therapie in Form einer 1,5%igen Creme für Patienten ab 12 Jahren mit NSV und Gesichtsbeteiligung zur Verfügung. Ruxolitinib‐Creme kann auf bis zu 10% der Körperoberfläche zweimal pro Tag appliziert werden, ohne systemisch relevante Wirkspiegel zu erreichen.^72^ Schwangerschaft und Stillzeit sind Kontraindikationen für die Anwendung von Ruxolitinib‐Creme, weshalb für die Dauer der Anwendung bei gebärfähigen weiblichen Personen eine Kontrazeption empfohlen wird. Wenn nach einem Jahr eine mindestens 25%ige Repigmentierung der behandelten Herde eingetreten ist, kann die Therapie fortgesetzt werden. Die Creme darf nicht auf Schleimhäute appliziert werden. Die Zulassung basiert auf einer placebokontrollierten Phase‐II‐Dosisfindungsstudie, in der sich die 1,5%ige Konzentration der Creme zweimal täglich als am stärksten wirksam erwies^73^ sowie auf zwei nachfolgenden placebokontrollierten Phase‐III‐Studien (TRueE‐V1 und TRueE‐V2) an insgesamt 674 Patienten.^74^ Nach 52‐wöchiger Behandlungszeit erreichten 50,3% der Patienten einen F‐VASI75, der als klinisch relevante Besserung angesehen wird. Circa 30% erzielten sogar einen F‐VASI90. Nach 52 Wochen Behandlung erreichten 51,1% der Patienten einen T‐VASI50. Beide Phase‐III‐Studien zeigten eine hohe Patientenzufriedenheit, gemessen an der *Vitiligo Noticeability Scale* und der *Color‐Matching Response*. Kopf‐ und Nackenbereich sprachen am besten auf die Ruxolitinib‐Creme an, gefolgt von Armen und Beinen, Stamm und zuletzt Händen und Füßen.^72^ Kein signifikanter Unterschied konnte zwischen Erwachsenen und Kindern gesehen werden; ebenso wenig hatten Dauer der Erkrankung, etwaige Begleitkrankheiten oder Vortherapien einen Einfluss auf das Therapieansprechen.^72^, ^74^ Die häufigsten unerwünschten Wirkungen waren akneartige Hautveränderungen (5,9% und 5,7%) und Juckreiz an den Applikationsstellen (5,0% und 5,3%); sie führten jedoch in keinem Fall zu einem Therapieabbruch.^74^ In den Verlängerungsstudien, in denen Ruxolitinib‐Creme für weitere 52 Wochen appliziert wurde, zeigte sich eine weitere Zunahme des Anteils von Patienten mit einem klinisch bedeutsamen F‐VASI75 auf 66%.^72^ Im Vergleich zu TCS und TCI ist die Therapie mit topischem Ruxolitinib vergleichsweise kostenintensiv. Die Gesamtwirtschaftlichkeit muss jedoch immer vor dem Hintergrund verfügbarer zugelassener gleichwertiger Alternativen und langfristiger Folgen wie der möglichen Entwicklung psychischer Komorbiditäten bei unzureichender Therapieeffizienz gesehen werden. Wie oben dargelegt, sind TCS für eine Langzeittherapie besonders im Gesichtsbereich ungeeignet und die Therapie mit TCI *off‐label*.

Mit der europaweiten Zulassung des JAK‐1/2‐Inhibitors Ruxolitinib steht seit April 2023 erstmals eine Erstlinien‐Therapie in Form einer 1,5%igen Creme für Patienten ab 12 Jahren mit NSV und Gesichtsbeteiligung zur Verfügung.

### Phototherapien

Heutzutage werden in erster Linie Schmalband‐UVB (NB‐UVB), außerhalb Europas auch noch die Photochemotherapie (PUVA) eingesetzt.^75^ Der Wirkmechanismus der Phototherapien beruht einerseits auf den pleiotropen immunmodulatorischen Effekten der UV‐Strahlung, andererseits auf deren ausgeprägter Stimulation des Pigmentsystems.^76^, ^77^, ^78^, ^79^ Neben einer Depletion von epidermalen Langerhans‐Zellen wirkt NB‐UVB der Aktivierung von Th17‐Zellen entgegen und führt zu einer Reduktion von IL‐17 und IL‐22 sowie einer stark verminderten JAK1‐Expression, zusätzlich kommt es zu einer Hochregulation von IL‐10, wodurch regulatorische T‐Zellen induziert werden. Analysen zirkulierender CD3^+^ CD8^+^ CD28^+^ T‐Zellen sowie zirkulierender CD4^+^ und CD8^+^ zentrale Gedächtnis‐T‐Zellen (T_CM_) zeigten signifikant verringerte Werte unter Phototherapie.^80^, ^81^ Gleichzeitig stimuliert die Bestrahlung mit NB‐UVB die Proliferation von Melanozyten und die Differenzierung und Migration von melanozytären Stammzellen in der äußeren Haarwurzelscheide und steigert die Melanogenese.

Der Wirkmechanismus der Phototherapien beruht einerseits auf den pleiotropen immunmodulatorischen Effekten der UV‐Strahlung, andererseits auf deren ausgeprägter Stimulation des Pigmentsystems.

### NB‐UVB‐Monotherapie

Die Ganzkörpertherapie mit NB‐UVB wird primär eingesetzt, um bei Patienten, bei denen aufgrund der Ausdehnung der Vitiligo eine Lokaltherapie nicht mehr angezeigt ist, eine Repigmentierung zu erzielen. Zusätzlich kann man durch eine regelmäßige Bestrahlung (2–3‐mal pro Woche über 6 Monate) bei Patienten mit aktiver und rasch progredienter Vitiligo die Krankheitsaktivität zum Stillstand bringen.^82^, ^83^ Das Ansprechen auf die NB‐UVB Phototherapie ist sehr variabel und hängt von zahlreichen Faktoren ab wie der UV‐Dosierung und Therapiedauer, Therapieadhärenz, Vitiligotyp, Hautphototyp, Erkrankungsdauer, Lokalisation der betroffenen Hautareale, Fehlen von Leukotrichie und psychischen Faktoren. Eine Metaanalyse zeigte, dass nach 12 Monaten Bestrahlung bei 56,8% der Patienten eine ≥ 50% Repigmentierung und bei 35,7% der Patienten eine ≥ 75% Repigmentierung der betroffenen Hautareale erzielt werden kann. Durch Kombination mit anderen Therapien (siehe unten) können diese Ergebnisse noch weiter verbessert werden. Die Phototherapie bei Vitiligo wird üblicherweise zwei‐ bis dreimal pro Woche durchgeführt und sollte alle 3 Monate evaluiert werden.^84^ Die Mindestdauer der Behandlung liegt bei 6 Monaten, da es länger als 3 Monate dauern kann, bis eine nennenswerte Repigmentierung eintritt.^85^, ^86^ Die Rezidivraten 12 Monate nach erfolgter NB‐UVB‐Therapie bewegen sich zwischen 21% und 45%, allerdings beruhen diese Daten auf sehr kleinen Fallzahlen.^88^, ^89^

Die Ganzkörpertherapie mit NB‐UVB wird primär eingesetzt, um bei Patienten, bei denen aufgrund der Ausdehnung der Vitiligo eine Lokaltherapie nicht mehr angezeigt ist, eine Repigmentierung zu erzielen.

### Kombinationstherapien

In vielen, zumeist kleinen und unkontrollierten Studien wurde versucht, die durch eine NB‐UVB‐Phototherapie erzielbare Repigmentierung durch Kombination mit topischen oder systemischen Therapien weiter zu verbessern. In Bezug auf die zusätzliche Applikation von topischen Kortikosteroiden zeigte eine randomisierte dreiarmige Studie (Mometasonfuroat Salbe vs. NB‐UVB‐Heimtherapie mit einem Handgerät vs. Kombination) an 517 Patienten (davon 370 auswertbar) nach einem Studienzeitraum von 9 Monaten lediglich eine leichte Überlegenheit der Kombinationstherapie.^90^ Die von einer Arbeitsgruppe beschriebenen synergistischen Effekte einer Pseudokatalase‐Creme und NB‐UVB^93^, ^94^ konnten in späteren Studien nicht bestätigt werden.^91^, ^92^ Im Gegensatz dazu scheint sowohl eine Kombination von NB‐UVB mit Vitamin‐D‐Derivaten^95^ als auch mit Tacrolimus^68^, ^96^, ^97^ wirksamer als eine NB‐UVB‐Monotherapie zu sein.

In zumeist kleinen und unkontrollierten Studien wurde versucht, die durch eine NB‐UVB‐Phototherapie erzielbare Repigmentierung durch Kombination mit topischen oder systemischen Therapien weiter zu verbessern.

Phototherapie mit zusätzlichen oralen Kortikosteroid‐Minipuls‐Gaben (siehe unten) kann ein schnelleres Sistieren der Krankheitsaktivität und eine höhere Repigmentierungsrate bewirken.^98^, ^99^, ^100^ In einer rezenten placebokontrollierten Vergleichsstudie von NB‐UVB gegen NB‐UVB plus Dexamethason Minipulse sprachen die Patienten in der Kombinationsgruppe zwar ebenfalls schneller an, nach 6 Monaten waren jedoch keine Unterschiede mehr im Ausmaß der Repigmentierung zwischen den beiden Gruppen feststellbar.^101^

Basierend auf der Bedeutung von oxidativem Stress in der Entstehung von Vitiligo wurden auch eine Reihe von Studien zur gleichzeitigen Verabreichung von systemischen Antioxidanzien mit einer NB‐UVB‐Phototherapie durchgeführt.^102^ Dabei konnte in randomisierten kontrollierten Studien sowohl für eine Mischung aus Liponsäure, Vitamin E, Vitamin C, ungesättigten Fettsäuren und Cystein^103^, Polypodium‐leucotomos‐Extrakt^104^, Vitamin E^105^ und Gliadinbiopolymer‐geschützte Superoxid‐Dismutase^106^ ein additiver therapeutischer Effekt nachgewiesen werden. Eine Monotherapie mit *Ginkgo biloba* zeigte in einer doppelblind randomisierten Studie einen krankheitsstabilisierenden und repigmentierungsfördernden Effekt.^107^ Zur Wirksamkeit von Ginko biloba in Kombination mit einer Phototherapie liegen keine Daten vor.

### Unerwünschte Wirkungen und Risiken

Die NB‐UVB‐Therapie ist bei korrekter Durchführung sicher und nebenwirkungsarm. Die häufigste akute Nebenwirkung ist ein leichtes bis moderates UV‐Erythem, das wie ein Sonnenbrand innerhalb von 24 Stunden nach der Bestrahlung auftritt und lediglich eine Dosisanpassung erfordert. Das photokarzinogene Risiko einer langzeitigen NB‐UVB‐Behandlung (> 100 Expositionen) muss krankheitsbezogen betrachtet werden. Es liegen mehrere Untersuchungen vor, denen zufolge, mit Ausnahme einer koreanischen Studie,^108^ Vitiligo‐Patienten ein geringeres Risiko für die Entstehung von nichtmelanozytärem und melanozytärem Hautkrebs aufweisen als Kontrollpersonen.^109^, ^110^, ^111^ Was das zusätzliche photokarzinogene Risiko einer NB‐UVB‐Therapie bei Vitiligo‐Patienten betrifft, zeigen die meisten Studien, dass die NB‐UVB‐Phototherapie nicht zu erhöhter Inzidenz von melanozytärem oder nichtmelanozytärem Hautkrebs führt.^112^, ^113^, ^114^ Lediglich in einer Arbeit aus Singapur wurde eine gering erhöhte Inzidenz von nichtmelanozytärem Hautkrebs bei NB‐UVB behandelten Vitiligo Patienten gefunden.^115^

Was das photokarzinogene Risiko einer NB‐UVB‐Therapie bei Vitiligo‐Patienten betrifft, zeigen die meisten Studien, dass die NB‐UVB‐Phototherapie nicht zu erhöhter Inzidenz von melanozytärem oder nichtmelanozytärem Hautkrebs führt.

### Gezielte Lichttherapien

Für die gezielte Lichttherapie kommen Bestrahlungsgeräte zur Anwendung, die hochintensives UVB‐Licht emittieren und bei segmentaler Vitiligo oder stabiler, kleinflächiger nichtsegmentaler Vitiligo angewendet werden.^116^ Diese Art der Lichttherapie wird auch adjuvant zu chirurgischen Verfahren eingesetzt, um die Therapieergebnisse zu optimieren^117^, ^118^ und kann in Kombination mit NB‐UVB für therapieresistente Läsionen eingesetzt werden.^119^ Die am häufigsten eingesetzten Geräte sind der Xenon‐Chlorid‐Excimerlaser und die nicht auf Lasertechnologie basierende Xenon‐Chlorid‐Excimerlampe, die beide Licht mit einer Wellenlänge von 308 nm emittieren.^120^, ^121^, ^122^

Für die gezielte Lichttherapie kommen Bestrahlungsgeräte zur Anwendung, die hochintensives UVB‐Licht emittieren und bei segmentaler Vitiligo oder stabiler, kleinflächiger nichtsegmentaler Vitiligo angewendet werden.

Im Vergleich zu einer NB‐UVB‐Behandlung tritt die Repigmentierung unter einer Excimer‐Therapie schneller und unter Einsatz geringerer kumulativer UV‐Dosen auf.^120^, ^123^, ^124^ Ein systematischer Review zeigte eine vergleichbare Effizienz von Excimer‐Laser und NB‐UVB hinsichtlich des Erreichens einer ≥ 75%‐ oder 100%‐Repigmentierung, wobei jedoch mit dem Excimer‐Laser eine höhere Rate an ≥ 50%‐Repigmentierung erzielt wurde.^125^ Excimer‐Laser und Excimer‐Lampe sind nach vorherrschender Datenlage in der Behandlung der Vitiligo gleich wirksam.^122^, ^126^, ^127^

Die Rezidivrate innerhalb von 1–3 Jahren nach einer Excimer‐Laser‐Therapie beträgt zwischen 2,6%–15%.^128^, ^129^ Eine Folgeuntersuchung von mit Excimer‐Laser behandelten Kindern nach durchschnittlich 3,4 Jahren zeigte lokalisationsabhängige Rezidivraten von 20% im Gesicht, 60% am Körper und 80% an den Händen.^130^ Die Kombination mit TCS oder TCI erhöht die Wirksamkeit der gezielten Lichttherapie und kann bei davor therapieresistenten Arealen ein Ansprechen ermöglichen.^97^, ^131^, ^132^, ^133^, ^134^, ^135^, ^136^, ^137^, ^138^ Zwei Übersichtsarbeiten zur Kombination von Excimer‐Licht und Vitamin‐D‐Analoga belegen keinen additiven Effekt einer topischen Vitamin‐D‐Anwendung.^95^, ^135^ In einer rezenten Netzwerk‐Metaanalyse von elf randomisierten Studien zeigte hingegen im Vergleich zu einer Monotherapie mit Excimer‐Laser die Kombination mit Tacalcitol, Calcipotriol oder einem *Cosmeceutical*, welches Superoxid‐Dismutase, Kupfer, Zink, Vitamin B12 und Calciumpantothenat enthielt, die größte Wahrscheinlichkeit einer 75%‐Repigmentierung.^139^ Diese Ergebnisse sind allerdings aufgrund geringer Fallzahlen und kurzer Studiendauern (≤ 4 Monate) sehr zurückhaltend zu interpretieren.

### Immunsuppressive Systemtherapien

Um die Aktivität der Vitiligo bei rasch progredienter NSV oder früher SV zu stoppen, sollte bei allen Patienten der Einsatz einer oralen Minipulstherapie mit Kortikosteroiden erwogen werden.^58^, ^63^ Die Therapie dient zur Stabilisierung einer aktiven Erkrankung und induziert üblicherweise keine Repigmentierung, daher ist eine zeitgleiche Einleitung der NB‐UVB‐Behandlung empfehlenswert (siehe oben). Empfohlen werden Betamethason (5 mg) oder Dexamethason (2,5–5 mg) an zwei aufeinanderfolgenden Tagen in der Woche, gefolgt von fünf therapiefreien Tagen, über 3 bis 6 Monate. Andere Kortikosteroide können in Äquivalenzdosen gegeben werden (Methylprednisolon in 5‐fach höherer Dosis, Prednison und Prednisolon in 6,25‐fach höherer Dosis als Dexamethason). In den vorliegenden nichtkontrollierten Studien wird über eine > 80%ige Stabilisierungsrate bei ausgedehnter rapid progressiver NSV durch die orale Minipuls (OMP)‐Therapie berichtet.^98^, ^140^, ^141^ Potenzielle unerwünscht Wirkungen wie Gewichtszunahme, Stimmungsschwankungen, akneartige Hautveränderungen, Schlafstörungen, menstruale Störungen, Hypertrichose, Wachstumsverzögerung bei Kindern und Immunsuppression müssen im Vorfeld mit den Patienten besprochen werden, sind aber in der Regel nicht ausgeprägt und führen nur selten zu einem Therapieabbruch.

Um die Aktivität der Vitiligo bei rasch progredienter NSV oder früher SV zu stoppen, sollte bei allen Patienten der Einsatz einer oralen Minipulstherapie mit Kortikosteroiden erwogen werden.

Andere immunsuppressive und immunmodulierende Substanzen wie Methotrexat, Ciclosporin, Azathioprin oder Minocyclin zeigten in kleineren nichtkontrollierten Studien uneinheitliche Effekte auf die Krankheitsaktivität. Keine dieser Therapien wird jedoch allgemein empfohlen und alle sind bei Vitiligo nur *off‐label* einsetzbar.^58^, ^63^ Für den Einsatz von Biologika gibt es derzeit ebenso keine Evidenz. Eine Therapie mit Tumornekrosefaktor (TNF)‐Inhibitoren erhöht laut einer großen koreanischen kontrollierten Langzeitstudie an 11 442 Patienten sogar das Risiko, eine Vitiligo zu entwickeln (*Hazard Ratio*: 1,99).^142^ Diese Ergebnisse werden durch eine WHO‐Pharmakovigilanz‐Datenbankstudie unterstützt, die neben TNF‐Inhibitoren auch *Checkpoint*‐Inhibitoren, Imiquimod, Alemtuzumab und Ustekinumab mit der Auslösung von Vitiligo in Verbindung bringt.^143^

Andere immunsuppressive und immunmodulierende Substanzen wie Methotrexat, Ciclosporin, Azathioprin oder Minocyclin zeigten in kleineren nichtkontrollierten Studien uneinheitliche Effekte auf die Krankheitsaktivität.

### Chirurgische Interventionen

Chirurgische Verfahren kommen primär bei SV und fokaler NSV, die auf konservative Therapie nicht ansprechen, zur Anwendung.^144^, ^145^ Als Kriterien für Stabilität der Vitiligo werden zumeist das Fehlen von Krankheitsaktivität in den vorausgegangenen 12 Monaten und das Fehlen eines Koebner‐Phänomens herangezogen (stabile Vitiligo).^59^, ^63^, ^146^ Man unterscheidet zwischen der autologen Transplantation von Gewebe (Vollhaut in Form kleiner Hautstanzen, Spalthaut oder mittels Saugblasen gewonnene Epidermis) und nichtkultivierter oder kultivierter melanozytärer oder epidermaler Zellsuspensionen.^145^, ^147^, ^148^, ^149^ Kultivierte Zellsuspensionen haben den Vorteil, dass durch die Expansion der Zellen wesentlich größere Areale behandelt werden können mit einer Spender‐Empfänger‐Ratio von bis zu 1 : 60,^150^ allerdings ist die Herstellung technisch anspruchsvoll, zeitaufwendig und kostenintensiv. Ein kommerziell erhältlicher Kit zur Herstellung autologer Zellsuspensionen (RECELL^®^) ist seit 2005 in Europa als Medizinprodukt auf den Markt und kann neben der Behandlung von Verbrennungswunden auch zur chirurgischen Intervention bei Vitiligo eingesetzt werden. In Amerika wurde dieser Kit erst im Juni 2023 von der FDA zur Behandlung von Patienten ab 18 Jahren mit stabiler Vitiligo zugelassen.

Chirurgische Verfahren kommen primär bei segmentaler Vitiligo und fokaler nichtsegmentaler Vitiligo, die auf konservative Therapie nicht ansprechen, zur Anwendung.

Die Deepithelisierung des Empfängerareals vor der Transplantation kann durch Dermabrasion, fraktionierter oder ablativer Laserbehandlung oder Saugblasentechnik erfolgen. Mitentscheidend für die Wahl der Transplantationsmethode ist die Lokalisation der Vitiligoareale, wobei bestimmte anatomische Regionen (Lippen, Lider, Genitalbereich, Finger und Zehen) chirurgisch herausfordernd sind.^144^, ^151^, ^152^, ^153^ Zu den möglichen Nebenwirkungen zählen je nach angewandter Technik postoperative Sekundärinfektionen, Farbdiskrepanzen zwischen dem repigmentierten Vitiligoareal und der umgebenden Haut^145^ ^.^ ^154^, Köbnerisierung, Narbenbildung im Spenderareal, eine kopfsteinpflasterartige Oberflächenstruktur im Empfängerareal nach der Transplantation von Hautstanzen und Milien.^145^, ^154^, ^155^

In einer rezenten Metaanalyse von 117 Studien wurde die Wirksamkeit der unterschiedlichen Transplantationsmethoden ausgewertet.^156^ Eine mehr als 90%‐Repigmentierung nach einer Behandlung wurde im Schnitt bei 72% der Patienten nach Spalthauttransplantation, 61,7% nach Transplantation von durch Saugblasentechnik gewonnener Epidermis, 56,8% nach Transplantation kultivierter Epidermalzellen, 47,5% nach Transplantation nichtkultivierter Epidermalzellen und 45,8% nach Transplantation von Hautstanzen erzielt, wobei die Ergebnisse in den unterschiedlichen Studien sehr heterogen waren. Entscheidend für einen guten Therapieerfolg sind neben der Expertise des Dermatochirurgen ein jüngeres Patientenalter, die Lokalisation der Empfängerareale und der Vitiligotyp (besseres Ansprechen von SV im Vergleich zu NSV).^157^ Obwohl ältere Studien nahelegen, dass eine adjuvante NB‐UVB‐Phototherapie des Empfängerareals das therapeutische Ergebnis weiter verbessert,^158^, ^159^, ^160^ konnte in der oben angeführten Metaanalyse dieser Effekt nicht bestätigt werden.

Entscheidend für einen guten Therapieerfolg sind neben der Expertise des Dermatochirurgen ein jüngeres Patientenalter, die Lokalisation der Empfängerareale und der Vitiligotyp.

Die Dauerhaftigkeit der erzielten Repigmentierung nach chirurgischer Intervention ist wenig untersucht. Erhöhte Rezidivraten wurden bei unzureichender Krankheitsstabilität, mangelhafter Repigmentierung nach der Transplantation und NSV gefunden.^154^, ^158^, ^161^ Nachbeobachtungen über 6 beziehungsweise 5 Jahre nach Transplantation nicht kultivierter epidermaler Zellsuspensionen zeigten eine unveränderte beziehungsweise immer noch ≥ 75%‐Repigmentierung bei mehr als der Hälfte der Patienten.^162^, ^163^ Da es nach einem chirurgischen Eingriff bis zu 2 Jahre dauern kann, bis sich eine maximale Repigmentierung einstellt, sollte bei ungenügendem Ansprechen entsprechend lang zugewartet werden, ehe eine erneute Transplantation durchgeführt wird.^163^

## SUPPORTIVE MAßNAHMEN

### Shared decision making

Bei einer gemeinsamen (partizipativen) Therapientscheidung bieten sich eine Reine von Hilfsmitteln an i (*shared decision making tools*), um krankheitsspezifische Hintergrundinformationen und inbesondere Informationen zu den Vor‐ und Nachteilen verfügbarer Therapien digital oder in Papierformat den Patienten zu geben. Diese können für die Therapieentscheidung nach gemeinsam definierten Therapiezielen (siehe oben) sehr hilfreich sein. Solche *shared decision making tools* sind für Vitiligo aber erst ansatzweise entwickelt.^164^

### Sonnenschutz

Aufgrund des Fehlens des photoprotektiven Eumelanins ist die depigmentierte Haut von Vitiligopatienten empfindlicher gegenüber UV‐Strahlung, weshalb die Anwendung hochpotenter äußerlicher Lichtschutzmittel (Sonnenschutzfaktor [SPF] ≥ 50) angeraten werden soll.

### Psychologische und psychosoziale Unterstützung

Der in der Dermatologie routinemäßig eingesetzte DLQI erlaubt bei Patienten mit Vitiligo ein erstes Screening auf psychosoziale Beeinträchtigung, ist aber nicht krankheitsspezifisch. Der VitiQoL ist im Gegensatz zum DLQI krankheitsspezifisch und somit für Vitiligo‐Patienten besser geeignet, liegt aber nicht in deutscher Sprache frei verfügbar vor. Die Verwendung kurzer Fragebögen wie *Generalized Anxiety Disorder‐2* (GAD‐2) und *Patient Health Questionnaire‐2* (PHQ‐2) erlaubt in der dermatologischen Routineversorgung ein einfaches dezidiertes Screening auf das mögliche Vorliegen einer Angsterkrankung und Depression und kann daher im Sinne eines holistischen Therapiemanagements der Vitiligo als systemische Entzündungserkrankung implementiert werden.^165^ Kontaktaufnahme mit Selbsthilfegruppen oder Selbsthilfeorganisationen (Deutscher Vitiligo‐Bund e.V. und Deutscher Vitiligo‐Verein e.V.) kann durch Austausch mit Gleichbetroffenen den Umgang mit der Erkrankung erleichtern.

Der in der Dermatologie routinemäßig eingesetzte DLQI erlaubt bei Patienten mit Vitiligo ein erstes Screening auf psychosoziale Beeinträchtigung. Der VitiQoL ist im Gegensatz zum DLQI krankheitsspezifisch und somit für Vitiligo‐Patienten besser geeignet.

### Camouflage

Camouflage ist ein wesentlicher Bestandteil der supportiven Maßnahmen bei Vitiligo. Eine Reihe von dermatokosmetischen Produkten sind verfügbar, die sich durch eine hohe Pigmentdichte und gute Haftung auszeichnen und eine exzellente Abdeckung der Vitiligoherde ermöglichen.^166^, ^167^ Selbstbräuner enthalten Dihydroxyaceton (DHA), das chemisch mit Proteinen im Stratum corneum reagiert. Die Haut bekommt dadurch einen 3–7 Tage anhaltenden goldbraunen Farbton. Der erzielte Farbton kann durch Variation der DHA‐Konzentration modifiziert werden, dennoch bestehen oft Farbdifferenzen zwischen der gesunden und der mit DHA‐behandelten Haut.^168^ Permanentes Make‐up ist eine weitere Option, insbesondere für therapieresistente Körperareale wie die Akren, Mamillen oder Lippen.^169^ Komplikationen wie Infektionen, allergische Reaktionen oder Narbenbildung sind selten.^170^ Der durch die Mikropigmente hervorgerufene Pigmentierungseffekt ist jedoch statisch, sodass im Verlauf der Vitiligo nicht immer zufriedenstellende Ergebnisse gewährleistet sind.^171^

Eine Reihe von dermatokosmetischen Produkten sind verfügbar, die sich durch eine hohe Pigmentdichte und gute Haftung auszeichnen und eine exzellente Abdeckung der Vitiligoherde ermöglichen.

### Depigmentierung

Eine Depigmentierung ist *ultima ratio* und wird fast nur bei Patienten mit dunklem Hautphototyp, subtotaler Vitiligo und großem Leidensdruck in Erwägung gezogen.^172^ Durch die Entwicklung neuer Therapien gerät das Ziel, verbleibende epidermale Melanozyten irreversibel zu zerstören, in den Hintergrund. Im deutschsprachigen Raum gibt es auch keine zugelassene Arzneimittelspezialität zur permanenten Depigmentierung. Eine fokale Restpimentierung kann durch Kryotherapie beseitig werden. Zumeist wird dafür aber der Monobenzylether von Hydrochinon (Monobenzone) angewendet, wodurch ein chemisch induziertes Leukoderm induziert wird.^173^ Die Behandlung erstreckt sich über Monate und führt zu einer deutlichen, aber oft nicht völligen Depigmentierung der behandelten Hautareale. Repigmentierungen in zuvor erfolgreich depigmentierter Haut können ebenfalls vorkommen.^174^ Konzentrationsabhängige Hautirritationen treten bei circa 50% der behandelten Patienten auf.^173^ Eine weitere Methode zur Destruktion von Melanozyten ist der Güte‐geschaltete Rubinlaser, der auch in Kombination mit chemischer Depigmentierung eingesetzt werden kann. ^175^, ^176^

Eine Depigmentierung ist *ultima ratio* und sollte nur bei Patienten mit dunklem Hautphototyp, subtotaler Vitiligo und großem Leidensdruck in Erwägung gezogen werden.

### Therapeutische Zukunftsperspektiven

Topische und systemische JAK‐Inhibitoren sind aktuell die am weitesten fortgeschrittene neue Substanzklasse in der klinischen Forschung bei Patienten mit Vitiligo. Ihre Wirksamkeit als Monotherapie oder in Kombination mit einer Phototherapie wird aktuell in zahlreichen klinischen Studien bei Patienten mit stabiler oder aktiver NSV evaluiert.

Topische und systemische JAK‐Inhibitoren sind aktuell die am weitesten fortgeschrittene neue Substanzklasse in der klinischen Forschung bei Patienten mit Vitiligo.

Zu den derzeit in klinischer Prüfung befindlichen JAK‐Hemmern gehören Tofacitinib, Ritlecitinib, Upadacitinib, Baricitinib, Povorcitinib und Cerdulatinib (https://classic.clinicaltrials.gov/). Ritlecitinib, ein JAK3/TEC‐Tyrosinkinase‐Inhibitor, besitzt bereits seit Ende 2023 in Deutschland eine Zulassung für ausgedehnte Formen der Alopecia areata. In einer umfangreichen placebokontrollierten Phase‐IIb‐Studie, in der 364 Patienten mit ausschließlich aktiver NSV und bis zu 50% BSA‐Beteiligung eingeschlossen wurden, führte Ritlecitinib in einer Dosis von 30 mg oder 50 mg pro Tag nach 24 Wochen zu signifikanten Verbesserungen des F‐VASI.^177^ Eine initiale vierwöchige Hochdosisphase von bis zu 200 mg Ritlecitinib hatte keinen zusätzlichen Effekt auf den primären Endpunkt (prozentuale Veränderung des F‐VASI gegenüber dem Ausgangswert). In der 24‐wöchigen Verlängerungsphase dieses RCT bewirkte Ritlecitinib (200 mg/50 mg) eine weiter zunehmende Repigmentierung des Gesichts. In einer Subgruppen‐Analyse von 65 Patienten aus der obigen Studie zeigte die mit Ritlecitinib behandelte Kohorte eine signifikante Reduktion in der Dichte des CD3^+^/CD8^+^‐Infiltrats und eine dosisabhängige Unterdrückung von Biomarkern der T‐Zell‐Aktivierung, verbunden mit einer Heraufregulierung von Melanozytenmarkern in der Haut.^178^ In einer aktuell durchgeführten multizentrischen Phase‐III‐Studie wird Ritlecitinib an Patienten mit ausgedehnter NSV unabhängig von der Aktivität der Erkrankung geprüft (https://classic.clinicaltrials.gov/), wobei die Messung klinisch relevanter Endpunkte (wie F‐VASI75) und aussagekräftiger PROM wesentliche zusätzliche Informationen vermitteln wird.

Upadacitinib, das seit 2021 für die Behandlung der atopischen Dermatitis in Deutschland zugelassen ist, zeigte gleichfalls dosisabhängige Wirkungen in einer randomisierten doppelblinden placebokontrollierten Phase‐II‐Dosisfindungsstudie bei 185 Patienten mit ausgedehnter NSV (mittlerer T‐VASI: 21,53 und mittlerer F‐VASI: 1,09).^179^ Die maximale prozentuelle Reduktion des F‐VASI lag bei –21,26 (Upadacitinib 11 mg pro Tag) und des T‐VASI bei –14,27 (Upadacitinib 22 mg pro Tag) nach 24 Wochen. Fortgesetzte Behandlung mit Upadacitinib zeigte auch nach 52 Wochen kein Plateau in der erreichten Repigmentierung. Aktuell wird eine multizentrische Phase‐III‐Studie mit diesem JAK1‐Inhibitor durchgeführt.

Basierend auf Fallberichten und kleineren Fallserien scheint eine UV‐Exposition die Wirkung topisch oder systemisch verabreichter JAK‐Inhibitoren zu verstärken.^180^, ^181^ In einer kleinen prospektiven kontrollierten Open‐Label‐Studie mit 33 Patienten, die mit 2 mg Baricitinib pro Tag über 16 Wochen behandelt wurden, zeigte sich in Kombination mit NB‐UVB eine ausgeprägtere Reduktion des F‐VASI und T‐VASI als unter einer NB‐UVB‐Monotherapie. Die alleinige Verabreichung von Baricitinib wurde allerdings nicht evaluiert.^182^

Ein weiterer Ansatz einer zukünftigen Therapie der Vitiligo besteht in der Verabreichung melanotrop wirkender Peptide allein oder in Kombination mit anderen Therapien. Afamelanotid (Nle5‐d‐Phe7‐α‐MSH) ist ein superpotentes synthetisches Analogon des α‐Melanozyten‐stimulierenden Hormons (α‐MSH), das in Form einer *Slow‐Release*‐Formulierung subkutan implantiert wird. Es ist seit 2015 für die Therapie der erythropoetischen Protoporphyrie zugelassen.^183^ α‐MSH und Melanokortin‐1‐Rezeptor (MC1R)‐aktivierende Agonisten haben neben ihrer klinisch eindeutigen pigmentierenden Wirkung zudem zytoprotektive, immunmodulierende und indirekte antioxidative Effekte,^184^, ^185^, ^186^ die bei der Behandlung der Vitiligo von Interesse sind. Eine additive monatliche Verabreichung von Afamelanotid erwies sich in zwei kleinen Studien einer alleinigen NB‐UVB‐Phototherapie in Bezug auf die Ansprechgeschwindigkeit und Repigmentierungsrate überlegen, allerdings war dieser Effekt auf dunkle Hautphototypen (IV–VI) beschränkt.^187^, ^188^ Ob Afamelanotid als Monotherapie bei Vitiligo‐Patienten mit Hautphototyp IV–VI wirksam ist, wird aktuell klinisch geprüft (https://classic.clinicaltrials.gov/). Kürzlich wurde über vielversprechende Wirkungen von Dersimelagon, dem ersten oral verfügbaren hochselektiven MC1R‐Agonisten, bei Patienten mit erythropoetischer Protoporphyrie berichtet.^189^ Dersimelagon befindet sich gegenwärtig in der klinischen Zulassungsphase bei dieser *orphan disease* und könnte sich als Non‐Peptid‐MC1R‐Agonist auch für zukünftige klinische Studien an Patienten mit Vitiligo als Therapie anbieten. Solche MC1R‐Agonisten wären möglicherweise speziell für Vitiligo‐Patienten mit dunkleren Hautphototypen geeignet.

Ein weiterer Ansatz einer zukünftigen Therapie der Vitiligo besteht in der Verabreichung melanotrop wirkender Peptide allein oder in Kombination mit anderen Therapien.

## DANKSAGUNG

Open access Veröffentlichung ermöglicht und organisiert durch Projekt DEAL.

## INTERESSENKONFLIKT

M.B. hat Beraterhonorare und/oder Vortragshonorare von Pharmafirmen (AbbVie, Incyte, MSD, Pfizer) erhalten, die Arzneimittel gegen Vitiligo entwickeln und vertreiben. Er erhält Forschungsgelder von Incyte zur Durchführung einer nichtinterventionellen Studie bei Patienten mit Vitiligo (VitiligoHealth). A.T. hat Beraterhonorare und Vortragshonorare von Incyte Biosciences Austria und Incyte International Sàrl erhalten.

## CME‐Questions – Lernerfolgskontrolle

1. Welche Aussage zur Ätiopathogenese der Vitiligo trifft zu?
  - Bei der NSV wurde eine Persistenz von Herpesviren in läsionaler Haut gefunden.
  - In der Haut von Patienten mit NSV sind die Spiegel reaktiver Sauerstoffspezies erhöht.
  - Bei der NSV sezernieren Keratinozyten Chemokine, die Th17‐Zellen mit einer IL‐17‐Signatur anlocken.
  - Es besteht eine gestörte Wundheilung bei der NSV.
  - Gedächtniszellen der Haut (T_RM_) werden bei der NSV durch IL‐4 aktiviert.
2. Welche Aussage zur NSV ist richtig?
  - Die Vitiligoherde sind asymmetrisch verteilt.
  - Schleimhäute sind niemals betroffen.
  - Die gemischte Vitiligo wird auch zur NSV gerechnet.
  - Das Gesicht ist niemals isoliert betroffen.
  - Der Verlauf hängt vom Hautphototyp ab.
3. Welche Aussage ist richtig?
  - Die SV ist häufiger als die NSV.
  - Die SV zeigt häufig Rezidive.
  - Die NSV kann häufig schon bei der Geburt vorliegen.
  - Die SV zeigt frühzeitig eine Leukotrichie.
  - Die NSV ist üblicherweise selbstlimitierend.
4. Welche Aussage ist **nicht** richtig? Zu den klinischen Zeichen einer aktiven Vitiligo zählen …
  - Inflammierte Vitiligo
  - Konfetti‐Läsionen
  - Positives Köbner‐Zeichen
  - Trichrom‐Vitiligo
  - Halo‐Naevi
5. Welche Aussage zur topischen Therapie der Vitiligo ist richtig?
  - Topische Calcineurinantagonisten sind topischen Kortikosteroiden überlegen.
  - Statt topischen Kortikosteroiden der Klasse IV sollten eher moderne Klasse‐III‐Präparate benutzt werden.
  - Topische Calcineurinantagonisten können zu Hautatrophie und Teleangiektasien im Gesicht verursachen.
  - Topische Kortikosteroide sollten nicht mit einer Phototherapie kombiniert werden.
  - Viele Studien belegen einen Zusatzeffekt von Antioxidanzien bei topischen Calcineurinantagonisten und Kortikosteroiden.
6. Welche Aussage zur aktuellen Therapie mit Ruxolitinib‐Creme ist korrekt?
  - Topisches Ruxolitinib ist für die SV zugelassen.
  - Kinder ab 12 Jahren mit NSV dürfen behandelt werden.
  - Mit Ruxolitinib‐Creme darf eine befallene Körperoberfläche von bis zu 20% behandelt werden.
  - Pruriginöse Hautveränderungen können als Nebenwirkung auftreten.
  - Die Therapiedauer ist auf 12 Monate begrenzt.
7. Welche Aussage zur Phototherapie der Vitiligo trifft zu?
  - Eine generalisierte aktive NSV sollte am besten mit einer gezielten Lichttherapie behandelt werden.
  - Eine NB‐UVB ‐Ganzkörper‐Phototherapie sollte nicht länger als 6 Monate durchgeführt werden.
  - Eine Kombination von NB‐UVB mit TCI verbessert signifikant die Repigmentierung an den Akren.
  - Bestimmte Antioxidanzien können in Kombination mit NB‐UVB die Repigmentierung verstärken.
  - Bei der NB‐UVB‐Ganzkörper‐Phototherapie sollte fünfmal pro Woche bestrahlt werden.
8. Welche Aussage zur Systemtherapie der Vitiligo trifft zu?
  - TNF‐Antagonisten können die Krankheitsaktivität stoppen.
  - Eine OMP‐Therapie der aktiven Vitiligo mit Dexamethason (2 x pro Woche mit 2,5–5 mg) wird üblicherweise über 3–6 Monate durchgeführt.
  - Systemische Kortikosteroide sollten nicht mit einer Lichttherapie kombiniert werden.
  - Systemische Kortikosteroide als Monotherapie führen bei aktiver NSV zu einer deutlichen Repigmentierung.
  - Ritlecitinib ist zur Therapie der aktiven oder rapid progressiven NSV seit November 2023 zugelassen.
9. Welche Aussage ist **nicht** richtig?
  - Der VitiQoL gilt als vitiligospezifisches PROM.
  - Der VES ist ein intuitiver Score zu Erfassung der Ausdehnung der Vitiligo in Prozent BSA und basiert auf Referenzbildern.
  - Der VES kann auch zuverlässig von Patienten mit Vitiligo angewandt werden (SA‐VES).
  - Der VASI errechnet sich aus Ausdehnung und dem jeweiligen Grad der Depigmentierung im Gesicht (F‐VASI) und am ganzen Körper (T‐VASI).
  - Der VETF‐Score ist zur Erfassung der Lebensqualität von Patienten mit Vitiligo der der geeignetste Score.
10. Welche Aussage zu den supportiven Maßnahmen bei Vitiligo ist richtig?
  - *Shared decision making* involviert sowohl Betroffene als auch Behandler und erlaubt eine gemeinsame Therapieentscheidung zugunsten einer verbesserten Compliance und Adhärenz.
  - Zur permanenten Depigmentierung wird eine 10% Hydrochinon‐Creme eingesetzt.
  - Entstigmatisierungsprogramme haben keinen Einfluß auf die psychosoziale Gesundheit erkrankter Personen.
  - Eine Therapie betroffener Patienten kann auch von Selbsthilfegruppen angeboten werden.
  - Grundsätzlich sollten alle Patienten mit Vitiligo eine Vitamin‐D‐Substitution erhalten, da deren Synthese in der Haut gestört ist.

Liebe Leserinnen und Leser, der Einsendeschluss an die DDA für diese Ausgabe ist der 31. October 2025.

Die richtige Lösung zum Thema Dermatologic diseases of the Breast and Nipple in Heft 05/2025 ist: 1c, 2c, 3e, 4a, 5d, 6a, 7e, 8a, 9d, 10c

Bitte verwenden Sie für Ihre Einsendung das aktuelle Formblatt auf der folgenden Seite oder aber geben Sie Ihre Lösung online unter http://jddg.akademie-dda. de ein.
